# In-Utero Exposure to Bereavement and Offspring IQ: A Danish National Cohort Study

**DOI:** 10.1371/journal.pone.0088477

**Published:** 2014-02-18

**Authors:** Jasveer Virk, Carsten Obel, Jiong Li, Jørn Olsen

**Affiliations:** 1 UCLA/Fielding School of Public Health, Department of Epidemiology, University of California Los Angeles, Los Angeles, California, United States of America; 2 Section for General practice, Department of Public Health, Aarhus University, Aarhus, Denmark; 3 Section for Epidemiology, Department of Public Health, Aarhus University, Aarhus, Denmark; Université de Montréal, Canada

## Abstract

**Background:**

Intelligence is a life-long trait that has strong influences on lifestyle, adult morbidity and life expectancy. Hence, lower cognitive abilities are therefore of public health interest. Our primary aim was to examine if prenatal bereavement measured as exposure to death of a close family member is associated with the intelligence quotient (IQ) scores at 18-years of age of adult Danish males completing a military cognitive screening examination.

**Methods:**

We extracted records for the Danish military screening test and found kinship links with biological parents, siblings, and maternal grandparents using the Danish Civil Registration System (N = 167,900). The prenatal exposure period was defined as 12 months before conception until birth of the child. We categorized children as exposed in utero to severe stress (bereavement) during prenatal life if their mothers lost an elder child, husband, parent or sibling during the prenatal period; the remaining children were included in the unexposed cohort. Mean score estimates were adjusted for maternal and paternal age at birth, residence, income, maternal education, gestational age at birth and birth weight.

**Results:**

When exposure was due to death of a father the offsprings' mean IQ scores were lower among men completing the military recruitment exam compared to their unexposed counterparts, adjusted difference of 6.5 standard IQ points (p-value = 0.01). We did not observe a clinically significant association between exposure to prenatal maternal bereavement caused by death of a sibling, maternal uncle/aunt or maternal grandparent even after stratifying deaths only due to traumatic events.

**Conclusion:**

We found maternal bereavement to be adversely associated with IQ in male offspring, which could be related to prenatal stress exposure though more likely is due to changes in family conditions after death of the father. This finding supports other literature on maternal adversity during fetal life and cognitive development in the offspring.

## Introduction

Several stressors during prenatal life have been shown to affect neurological development and cognitive outcomes in the offspring [1]. Anxiety, depression, natural disasters, and domestic violence have been used as markers of severe stress and linked to the development of autism, schizophrenia [2], emotional/behavioral problems [3]–[5], and reduced cognitive abilities in the offspring [6]. However, the range and severity of stressors that predict adverse outcomes has a wide range and includes minor stressors such as daily hassles, and more severe trauma such as anxiety, depression and bereavement [7]. Both maternal temperament and access to ameliorating resources may moderate the biological response of stress but these factors are difficult to measure, and direct measurement of stress such as allostatic load may not be feasible due to either ethical or economic reasons.

In general, the human placental enzyme 11β-HSD2 that converts active glucocorticoids to inactive metabolites [8] and reduces the risk of elevated levels of glucocorticoids to the fetus [9] appears around 20–24 weeks of gestations and remains stable until late gestation. A reduction in the 11β-HSD2 enzyme has been found in a range of pathological conditions, which may involve fetal exposure to elevated cortisol [10], [11]. A reduction in 11β-HSD2 may also affect other aspects of placental structure and function which affects fetal development. It has been shown that 11β-HSD2 knock-out mice had smaller placenta with reduced capillary surface area, volume and density, and a decrease in glucose transport [12]. There is also evidence that suggests the emotional state of the mother may alter the placenta independent of cortisol in a way that affects fetal neurodevelopment. Prenatal anxiety in late pregnancy has been associated with reduced blood flow in umbilical or uterine arteries and altered fetal cerebral circulation [13].

Prenatal bereavement has been associated with several neurological outcomes, such as attention/deficit/hyperactivity disorder [14], epilepsy [15], febrile seizures [16], and cerebral palsy [17]. Bereavement is classified as one of the most severe stress inducing life events, especially when the cause is due to unexpected or unforeseen events [18], the loss is of one's spouse or the loss of a child [19]. Intelligence is a life-long trait that has powerful influences on educational attainment, occupational success, lifestyle, adult morbidity and life expectancy [20]. Prior research demonstrates a link between prenatal stress and adverse neurodevelopment. We hypothesized that prenatal exposure to bereavement would adversely affect IQ, measured in early adulthood. Our primary aim in this analysis was to examine the association between prenatal bereavement measured as exposure to death of a close family member and IQ scores of young adult Danish males completing a military cognitive screening examination at or shortly after 18-years of age. We included relationship to the deceased and also assessed deaths due to unexpected events in our analysis. Our expectation was that the most severe stressor; loss of an elder child – sibling or loss of a spouse would have the strongest adverse effect on the IQ of a developing fetus.

## Methods

We extracted N = 241,222 individual records from the Danish Conscript Register. All test-takers were born between January 1^st^ 1976 and December 31^st^ 1993. Among these recruits, we found kinship links for N = 205,393 records using the Danish Civil Registration System (CRS). We eliminated missing and invalid test scores as well as test scores of female conscripts (N = 6713), due to a lack of sufficient sample size. Since 1968, all live born children and new residents in Denmark are assigned a unique civil personal registration number, allowing for accurate linkage at the individual level between all national registries. Kinship links were made for N = 167,900 males with their biological parents (mother and father), siblings, and maternal grandparents. Information on birth outcomes, such as gestational age at birth and birth weight, were obtained from the Danish Medical Birth Registry which has been computerized since 1973. Annual information on maternal education, residence, and income was retrieved from the Fertility Database at Statistics Denmark, available since 1979. These variables were treated as time dependent for each pregnancy for women who contributed to the cohort more than once. The date of conception was estimated by subtracting gestational age from the date of birth. We defined the prenatal exposure period as 12 months before conception until the birth of the child. We categorized children as exposed to bereavement during prenatal life if their mothers lost an elder child, husband, parent or sibling during the prenatal period; the remaining children were included in the unexposed cohort.

IQ was estimated using the Danish military draft board's Børge Priens Prøve (BPP) test [21]. This test is administered to all Danish men 18-years of age, with the exception of individuals providing medical documentation for disqualifying conditions for military service which include epilepsy, spinal osteochondrosis, diabetes and severe asthma. The test takes less than one hour to complete, has four sub-tests (letter matrices, verbal analogies, number series and geometric figures) and a total score ranging from 0 to 78. The BPP has remained unchanged since its first use in the 1950s, including the pencil/paper format. The total BPP score has a correlation of 0.82 with the full scale IQ of Wechsler's Adult Intelligence Scale, indicating that the BPP is a suitable measure of general intelligence [22].

We compared mean IQ scores among individuals exposed to bereavement and defined the unexposed group as those pregnancies unaffected by prenatal bereavement. F-tests provided by SAS version 9.1.3 PROC MIXED were used to evaluate differences between exposure groups. The models used minimum variance quadratic unbiased estimation method and a compound symmetry covariance structure. To control for the correlation between siblings, the models included a repeated subject statement with an identifying variable for the subjects' mother. All mean score estimates were adjusted for maternal and paternal age at birth (≤18, 19–34, 35–40, 41+); residence (Copenhagen, cities with over 100,000 inhabitants, and other); income (1^st^, 2^nd^, 3^rd^, and 4^th^ quartile); maternal education (primary: mandatory educational which is completed before 16 years of age; secondary: consists of an additional 2–4 years of training usually between 15–20 years of age, high: additional post-secondary education); gestational age at birth (<32 weeks, 32–36 weeks, ≥37 weeks); and birth weight (<1500 grams, 1500–2499 grams, ≥2500 grams). Age, residence, maternal income and parental cohabitation were treated as time-dependent variables.

To examine the relation between type of bereavement and IQ test scores, we categorized exposed children by relationship to the deceased. We further categorized the cause of death by traumatic death (traumatic causes: ICD-8 codes 7950–7959, ICD-10 codes R95-R97; motor vehicle accidents: ICD-8 codes 8100–8230, ICD-10 codes V01-V89; suicide: ICD-8 codes 950–959, ICD-10 codes X60-X84; and other accidents and violence: ICD-8 codes 800–807, ICD-10 codes V90-V99, W00-X59, X85-Y89); and death from other causes. This study was approved by the Danish Data Protection Agency (Ref no. 2008-41-2680) and the Ethics Committee for Central Jylland in Denmark (Ref no. M-20100252). Individual written consent was not obtained from study participants as we had no record linking the subjects to individual identifying information.

## Results

Almost all maternal demographic variables - maternal age, paternal age, household income, maternal education at birth, maternal residence, birth weight and gestational age were associated with exposure *(see* *table 1* *)*. Approximately 2.8 percent of our cohort was exposed to maternal bereavement prenatally (N = 4651) and among these exposed pregnancies 0.4 percent of deaths were due to traumatic events. We observed a lower mean IQ test score among all exposed groups, though only some groups reached both clinical and statistical significance. Compared to the standard deviation of the total sample (9.7), the 4.2 adjusted BPP IQ point different amounts to 0.43 standard deviation. A typical IQ scale has a mean of 100 and a standard deviation of 15, thus 0.43 BPP standard deviation corresponds to about 6.5 standard IQ points. The adjusted mean differences in BPP IQ scores transformed to a typical IQ scale are also presented in table 2. We found a clinical and statistical difference in mean IQ scores when exposure was due to death of a father (6.5 standard IQ point difference). Similarly, those exposed to death of an uncle/aunt also had lower mean IQ scores (3.0 standard IQ points) but this difference was not statistical and had marginal clinical significance. Furthermore, we did not find a significant clinical and/or statistical difference in mean IQ scores among those who were exposed to a sibling or maternal grandparents' death. We conducted additional analyses on timing of breavement by grouping exposed mothers by deaths occuring during preconception, and during the first, second or third trimesters and found no pattern or vulnerable exposure periods. Almost all adjustment variables included in the model - maternal age at birth, maternal education, place of birth, income, gestational age, and birth weight) with the exception of paternal age were significantly associated with offspring IQ scores for all models. The difference between the exposed and unexposed mean BBP IQ scores are also presented in table 2.

**Table 1:** Maternal characteristics at time of birth of all Danish men completing military entrance exam from 1976–1993, by exposure status to prenatal maternal bereavment* §.

	Total Cohort (N = 1,878,246)		Exposed (N = 45,302)		Unexposed (N = 1,832,944)	
	[N = 167,900]		[N = 4,651]		[N = 163,249]	
	N	(%)	N	(%)	N	(%)
**Maternal age**						
≤18	2550	1.5	33	0.7	2517	1.5
19–34	150634	89.7	4281	92.0	146353	89.7
35–40	13546	8.1	326	7.0	13220	8.1
41+	1170	0.7	11	0.2	1159	0.7
**Paternal age**						
≤18	417	1.9	48	1.1	369	2.0
19–34	128549	79.8	3523	79.4	125026	79.8
35–45	26979	16.8	805	18.1	26174	16.7
46+	2450	1.5	64	1.4	2386	1.5
**Maternal Income**						
1st quartile	8530	5.1	149	3.2	8381	5.2
2nd quartile	29455	17.6	647	13.9	28808	17.7
3rd quartile	74642	44.6	2245	48.3	72397	44.5
4th quartile	54791	32.7	1609	34.6	53182	32.7
**Maternal Education**						
Primary	60874	36.4	1769	38.0	59105	36.3
Secondary	53745	32.1	1504	32.3	52241	32.1
High	47794	28.5	1331	28.6	46463	28.5
**Maternal Residence**						
Copenhagen	40619	24.3	1080	23.2	39539	24.3
Big cities*	19812	11.8	512	11.0	19300	11.9
Other	106907	63.8	3059	65.8	103848	63.8
**Birth Weight (grams)**						
<1500	9381	5.6	82	1.8	9299	5.7
1500–2500	5988	3.6	202	4.3	5786	3.5
>2500	152531	90.9	4367	93.9	148164	90.8
**Gestational age (weeks)**						
<34	10181	6.1	108	2.3	10073	6.2
34–36	7117	4.2	233	5.0	6884	4.2
>37	150602	89.7	4310	92.7	146292	89.6

*difference between expossure groups for all characteristics yielded chi-squared test p-values less than 0.0122.

§ missing values for paternal age (n = 6805), income (n = 482), school (n = 411), residence (n = 411).

**Table 2:** Mean IQ scores, and difference between exposed and unexposed IQ scores by prenatal maternal bereavement* §.

All Deaths			Maternal	Maternal
	Father	Sibling	Grandparent	Uncle/Aunt
**Exposed sample**	n = 46	n = 707	n = 3829	n = 94
**Mean BPP Score**				
**Unexposed (unadjusted)**	41.8	41.8	41.8	41.8
**Exposed (unadjusted)**	36.6	41.3	41.7	39.5
**Unexposed (adjusted)**	39.6	39.6	39.6	39.6
**Exposed (adjusted)**	35.4	39.5	39.2	37.7
**Difference in adjusted means**	4.2	0.2	0.4	1.9
**Difference in adjusted means (Std IQ scale)**	6.5	0.2	0.6	3.0
**P-value, F-test**	0.01	0.65	0.01	0.06
**Traumatic Deaths** ‡				
**Exposed sample**	28	207	345	60
**Mean BPP Score**				
**Unexposed (unadjusted)**	41.8	41.8	41.8	41.8
**Exposed (unadjusted)**	36.6	40.9	41.1	39.1
**Unexposed (adjusted)**	39.6	39.6	39.6	39.6
**Exposed (adjusted)**	35.7	39.4	39.0	37.6
**Difference in adjusted means**	3.9	0.2	0.7	2.0
**Difference in adjusted means (Std IQ scale)**	6.1	0.4	1.0	3.1
**P-value, F-test**	0.06	0.72	0.18	0.12

*adjusted for maternal and paternal age at birth, income, residence (urban/rural), maternal education at birth, gestational age, and birth weight.

‡ traumatic deaths classified as deaths due to: unexpected causes, motor vehicle accidents, suicide, other accidents, violence, and death from other causes.

§ observations with missing values are not included in the adjusted analyses.

## Discussion

We found prenatal bereavement due to death of a father was adversely associated with IQ scores in early adulthood among Danish men completing the military screening exam. This association remained even after stratifying on deaths due to traumatic causes – which may partially control for background factors associated with reduced cognitive abilities. The lack of association detected when bereavement was due to a sibling death may however indicate that prenatal stress does not modify IQ in the offspring. It is possible that death of a father leads to loss in household income and overall socio-economic status, factors both associated with IQ, and that the observed association is due to post-natal changes rather than a prenatal gestational insult. Alternatively, the lack of association for bereavement due to a sibling death may be due to overcompensation in caring for a surviving child - although there is no current literature that supports this hypothesis. In either case, our findings support that IQ has more than genetic determinants.

Several prenatal factors have been identified with offspring intelligence. A study on cognitive function found that infants born small for gestational age had lower IQ scores than their non-SGA counterparts [23]. Similarly, gestational age and birth weight has been associated with school performance [24] and IQ test scores in young adults [25]. Family dynamics such as birth order has also been associated with cognitive development. Earlier studies found birth order to be a strong predictor of IQ though more recent literature suggests that this association disappears once family size is accounted for [26], [27]. Maternal intelligence is an important predictor of offspring intelligence, more so than race, education, age, poverty status, smoking, the home environment, or the child's birth weight or birth order [28]. This has been shown even when breastfeeding is accounted for, a factor often positively associated with IQ in the offspring [28].

Adverse fetal exposures during gestation and subsequent programming of the fetus has been established in animal models, and human studies utilizing data from natural occurrences/disasters are expanding research in this area. The hypothesis that stress increases maternal cortisol (corticosterone in rodents) which causes a range of short and long-term adverse health effects in fetal development is well documented in the literature [29]–[33]. Several animal studies have shown an effect of prenatal stress in programming the function of the HPA axis in the offspring, which often results in a prolonged and greater cortisol/corticosterone response to stressors later in life [34], [35], although humans may be better protected. The role of increased cortisol in the human fetal brain has been examined in microarray analyses and demonstrated that an increase in cortisol exposure affects the expression of over a thousand genes in fetal brain cells [36]. Furthermore, substantial resilience in complex human biological models is indicated by the modest association seen in epidemiologic studies.

The main strengths of our study are the large cohort size and high quality of exposure data. Information in the CRS has been made available for research purposes by the Danish Data Protection Agency [37]. Parental links for individuals born in Denmark since 1969 are considered accurate; and Danish legislation requires all legal address changes be submitted to the CRS [38]. All deaths in this study were identified via the Death Registry which reports high validity on vital status [39]. One of the main limitations in this study is the lack of inclusion of other sources of stressors and baseline measures of biological stress responses and measures of allostatic load. We were also not able to include mothers' mental state or support networks in the analysis, which are likely to attenuate the effect of bereavement.

However, it is unlikely that exposure contrast between the bereaved and non-bereaved exposure groups in our cohort is insubstantial. Furthermore, longitudinal sampling to estimate allostatic load would be impossible due to legal, logistic and economic reasons. Another possible limitation is the possibility of the study being over-powered. In a sub-analysis, we attempted to address this issue by using pregnancies exposed to a grandparents' death as controls and found no significant difference in interpretation of the results. The difference in standard IQ points using a control group bereaved to maternal grandparents was 5.7, only slightly lower than the 6.5 point difference when comparing to the unexposed group (p-value remained unchanged).

A concern often raised with the validity of the Børge Priens Prøve is the motivation for optimal test performance in males reluctant to be drafted. Since some men try to avoid service in the military, it is expected that this may negatively influence test performance. However, it is difficult to avoid military service by deliberately performing poorly during IQ testing. A study of 2,236 men found that the small minority of men expressing negative attitudes of military participation had in fact higher educational status and that this sentiment was associated with a desire for uninterrupted pursuit of higher education. These individuals also performed better on the IQ test [21], [40]. The reliability of the BPP test has also been recently evaluated in a small group (n = 105) of military personnel showing a correlation of 0.77 between test taken at the age of 18 and 22 years of age [40].

In our study we found maternal bereavement by death of the father to be adversely associated with intelligence in male offspring. This finding supports other literature on maternal adversity during fetal life and cognitive development in the offspring. However, it is also possible that the causes of the association are related to a change in social conditions rather than fetal programming of the HPA axis due to stress, since we found no clear association when bereavement was due to loss of a child, or other relationship status. Our results are limited to male offspring and a replication of this study is warranted among females.
